# Prostaglandin E2 EP4 Receptor Activation Attenuates Neuroinflammation and Early Brain Injury Induced by Subarachnoid Hemorrhage in Rats

**DOI:** 10.1007/s11064-016-2168-6

**Published:** 2017-02-27

**Authors:** Jie Xu, Zhen Xu, Ai Yan

**Affiliations:** 1 Department of Neurosurgery, Huzhou Central Hospital, 198 Hongqi Lane, Huzhou, 313003 China; 2 Department of Neurosurgery, First Affiliated Hospital of Zhejiang Chinese Medicine University, 54 Youdian Lane, Hangzhou, 310006 China

**Keywords:** EP4 receptor, Inflammation, Early brain injury, Subarachnoid hemorrhage

## Abstract

Activation of E prostanoid 4 receptor (EP4) shows neuroprotective effects in multiple central nervous system (CNS) lesions, but the roles of EP4 receptor in subarachnoid hemorrhage (SAH) are not explored. This study was designed to research the effects of EP4 modulation on early brain injury (EBI) after experimental SAH in rats. We found that the administration of EP4 selective agonist AE1-329 significantly improved neurological dysfunction, blood brain barrier (BBB) damage and brain edema at 24 h after SAH. Furthermore, AE1-329 obviously reduced the number of activated microglia and the mRNA and protein levels of pro-inflammatory cytokines, and increased Ser1177 phosphorylated endothelial nitric oxide synthase (Ser1177 p-eNOS). Moreover, AE1-329 significantly reduced the number of TUNEL-positive cells and active caspase-3 in cortex after SAH. The EP4 selective antagonist AE3-208 was also administrated and the opposite effects were achieved. Our results indicate that activation of EP4 protects brain from EBI through downregulating neuroinflammation reaction after SAH.

## Introduction

Subarachnoid hemorrhage (SAH) is one of the most devastating cerebrovascular diseases with high morbidity and mortality, and often results in long lasting neurological disability for survivors [1]. Despite the advances in neurointensive care, the underlying mechanisms of SAH-induced secondary brain injury are still incompletely understood. During the past decades, research efforts have been centered around vasospasm which is considered to be the most important factor for delayed neurological deficit [2, 3]. However the long lasting failure of anti-vasospastic treatments to improve outcome of SAH in most clinical trials [4] has brought into focus the significance of a more recently found pathological phenomenon which named early brain injury (EBI) [5]. EBI refers to the global brain injury which starts immediately after SAH and lasts 72 h until the development of vasospasm [6, 7]. Evidences suggest that SAH-induced EBI is not only responsible for the initial signs and symptoms on admission, but also blamed for the delayed neurological deterioration which is associated with vasospasm and poor long-term prognosis [8, 9]. The underlying pathophysiological mechanisms of EBI after SAH are not definitely clarified to date. It is generally accepted that neuroinflammation plays a critical role in the happening and progressing of EBI after SAH [10–12].

Microglial cell is the resident macrophage of the central nervous system (CNS). After SAH, especially in acute stage, activated microglias provoke excessive secretion of pro-inflammatory cytokines contributing to the development of brain edema, disruption of blood–brain barrier (BBB), and secondary neuronal injury after SAH [5]. The pro-inflammatory cytokines secreted by activated microglial cells include interleukin-6 (IL-6), interleukin-1 beta (IL-1β) and tumor necrosis factor alpha (TNF-α) [13] all of which have been found to be increased early after SAH and strongly linked to the brain injury in both patients and animals [14–16]. Thus, modulating the inflammatory cytokine secretion of activated microglial cells might be a potential strategy for the treatment of EBI after SAH.

The lipid signaling molecule prostaglandin E2 (PGE2), which is one of the most typical downstream products of arachidonic acid (AA) by cyclooxygenases-1 and 2 (COX-1 and 2), is a well-established modulator of inflammatory responses in a variety of CNS and peripheral injury models [17]. PGE2 modulates inflammatory responses through activating four distinct G protein-coupled receptors (GPCRs) named E prostanoid 1–4 receptors (EP 1–4 receptors), which exhibit divergent cellular expression profiles, desensitization kinetics and signaling cascades [18, 19]. Among them the EP4 receptor is emerging as the most promising and versatile one and the effects of anti-thrombosis, anti-inflammation and vasodilation have been proposed for it [20]. In vivo and in vitro studies have proved that activation of EP4 receptor by exogenous EP4 selective agonist suppresses microglial inflammatory response to Aβ_42_ peptides and lipopolysaccharide while conditional deletion of microglial EP4 conversely increases inflammatory gene expression [21, 22]. A broad variety of experimental neuropathological models associated with inflammation are also alleviated by the activation of EP4 receptor, including cerebral ischemia [23, 24], hypoxic-ischemic encephalopathy (HIE) [25], neurotoxicity induced brain injury [26] and Alzheimer’s disease [21].

The roles of EP4 receptor in EBI after SAH are currently unknown. Based on these prominent studies, it is reasonable to deduce that the activation of EP4 receptor might suppress microglial activation as well as microglia-induced neuroinflammation and thus play a protectional role in EBI after SAH.

In this study, we investigated the effects of EP4 receptor activation on the microglial activation, neuroinflammation and EBI after SAH by selectively using EP4 receptor specific agonist or antagonist in a rat model.

## Materials and Methods

### Animals

Experimental animal was male Sprague–Dawley rats (280–350 g), afforded by animal experimental center of Zhejiang Chinese Medicine University. The use and care of animals employed in our model were approved by the Animal Care and Use Committee of Zhejiang Chinese Medicine University, in accordance with all relevant laws of China.

All animals were kept in a room with controlled temperature of 23 ± 1 °C at 12-h dark/light cycle and fed with standard food and water ad libitum.

### Rat Model of SAH

An endovascular perforation method was used to produce the model of experimental SAH according to previous studies [27, 28]. Briefly, after anesthetizing with the mixture of 3% isoflurane in 70%/30% medical air/oxygen, the right external carotid artery was transected distally and then reflected caudally in line with the internal carotid artery. A blunted monofilament nylon suture of 4-0 was inserted into the right external carotid artery stump and then carefully advanced into the internal carotid artery until a resistance was felt, approximately 20–24 mm from the common carotid artery bifurcation. The suture was advanced for another 3 mm to perforate the right internal carotid artery close to the bifurcation with the middle cerebral artery to produce SAH. Sham-operated animals were suffered the similar procedures except the arterial perforation.

After operation, the rats were returned to their cages with the room temperature kept at 23 ± 1 °C. Physical cooling (ice bag) was used to keep the rectal temperature at 37 ± 0.5 °C when required.

### Drug Administration

The EP4 receptor selective agonist AE1-329 and antagonist AE3-208 were from Ono Pharmaceuticals Co (Japan). Their specificity for the EP4 receptor has been proved previously [29, 30]. AE1-329 (0.3 mg/kg) was subcutaneously injected (SC) [23]. In several models of cerebral diseases, AE3-208 is administrated per os to reach an effective concentration in brains [31, 32]. Based on these studies, we administrated AE3-208 (0.3 mg/kg) subcutaneously. AE1-329 and AE3-208 were dissolved in a solution consisting of 5% *N*-methyl pyrrolidone (NMP) and 5% solutol HS in saline, and each rat was subcutaneously injected with 2 ml/kg AE1-329, AE3-208 or vehicle (5% *N*-methyl pyrrolidone (NMP) and 5% solutol HS in saline).

All drugs were administrated immediately after SAH and were repeated 12 h post SAH with the same dose.

### Assessment of SAH Grade, Mortality and Neurological Score

The methods of SAH grade [33] and neurological score [34, 35] evaluation were described by previous studies and all evaluations were performed by an experimenter blinded to this study.

Twenty-four hours after SAH, rats were sacrificed after anesthetization and brains were removed immediately for the measurement of SAH grades. SAH grade was evaluated according to the amount of subarachnoid blood clots distributed in the six segments of basal cistern: grade one (scores = 0), no observable subarachnoid blood clots; grade two (scores = 1), minimal subarachnoid blood clots; grade three (scores = 2), moderate subarachnoid blood clots with recognizable arteries; and grade four (scores = 3), subarachnoid blood clots covering all arteries. Possible scores ranged from 0 to 18 were calculated by the total scores from all six segments. The animal’s neurological deficits were evaluated by six aspects: spontaneous activity (scores ranged from 0 to 3), reaction to side stroking (scores ranged from 1 to 3), vibrissae touch (scores ranged from 1 to 3), forepaw outstretching (scores ranged from 0 to 3), climbing (scores ranged from 0 to 3), limb symmetry (scores ranged from 0 to 3), and beam walking ability which refers to the walking distances on a wooden beam for 1 min (scores ranged from 0 to 4). The minimum score of neurological deficits was 2 and the maximum was 22; a higher score represent better neurological performance. Mortality was calculated at the same time.

### Assessment of Brain Edema

Twenty-four hours after SAH, rats were sacrificed after anesthetization and entire brains were removed immediately for the measurement of water content [36]. Water content of brain was determined by the formula (wet weight − dry weight)/(wet weight) × 100%. The wet weight of brains was obtained by the weight of freshly removed brains, then the wet brains were kept in an oven for 72 h at 100 °C and weighed to obtain dry weight.

### Blood Brain Barrier Permeability

BBB permeability was determined by Evans blue (EB) extravasation 24 h after SAH. Briefly, 2% Evans blue dye was injected in 2 min into the right femoral vein at a dose of 2 ml/kg, allowing the dye to circulate for a total of 60 min [37]. Animals were re-anesthetized and subjected to transcardial perfusion with phosphate buffered saline (PBS) to remove intravascular EB dye, then entire brains were removed and homogenized in phosphate buffered saline. Trichloroacetic acid was then added to precipitate protein. After overnight incubation at 4 °C, samples were centrifuged and the resulting supernatants were measured for absorbance of EB at 620 nm using a spectrophotometer.

### TUNEL Staining

Twenty-four hours after SAH, rats were anesthetized and perfused transcardially with 4% paraformaldehyde. Brains were separated and fixed in 4% paraformaldehyde for 24 h and dehydrated with gradient sucrose solution. Consecutive coronal sections of 10 μm were cut from bregma with 200 μm intervals. A total of 8 sections in right hemisphere of brain (perforation side) were obtained for terminal deoxynucleotidyl transferase-mediated dUTP-biotin nick end labeling (TUNEL) staining (Roche Diagnostics, Mannheim, Germany). DAPI staining was performed according to the instructions. The images were viewed with an EVOS-fl digital inverted fluorescent microscopy and five non-overlapped vision fields were randomly chosen for observation in each section. The number of TUNEL positive cells was counted with Image Pro Plus 6.0 Software (MediaCybernetics, Bethesda, USA). The mean number of TUNEL/ DAPI double positive nuclei in the five views was taken for the apoptotic index of each section and the apoptotic index for each animal was calculated by the final average percentage of double positive cells of the 8 sections.

### Western Blot

The cortex and cerebral microvessels of right hemisphere were separated for western blot analysis. Samples were incubated with protease inhibitor (Roche) then diluted with sample buffer. Samples were supplemented with 2% beta-mercaptoethanol and 50 mM DTT and boiled for about 5 min. Proteins were separated on tris-glycine 4–15% acrylamide gels and transferred to PVDF membranes soaked in 5% nonfat milk in PBS-Tween 20 (0.05%) for 2 h. The proteins of total caspase-3, cleaved caspase-3, Ser1177 phosphorylated endothelial nitric oxide synthase (Ser1177 p-eNOS) and β-actin were tested by rabbit monoclonal antibody against caspase-3 (Santa Cruz, 1:500), rabbit polyclonal antibody against cleaved caspase-3 (Biorbyt, 1:500), rabbit polyclonal antibody against Ser1177 p-eNOS (Invitrogen, 1:400) and rabbit polyclonal antibody against β-actin (Santa Cruz, 1:500) respectively. Immunoreactivity was tested by incubation with secondary HRP-coupled antibody for 1 h at room temperature followed by the ECL plus reagent (Santa Cruz). The densities of the bands were determined by the MiVnt image analysis system (Bio-Rad, Carlsbad, CA, USA).

### Immunofluorescence Staining

Consecutive coronal sections of 10 μm were cut from bregma with 200 μm intervals. A total of eight sections in right hemisphere (perforation side) were obtained for immunofluorescence staining. The sections were incubated with rabbit polyclonal antibody against Iba-1 (Santa Cruz, 1:300) at 4 °C overnight, then followed by incubating with Dylight 488-conjugated goat anti-rabbit antibody (Jackson ImmunoResearch, 1:1000). DAPI was used for nuclear staining. Sections viewed under the confocal microscope (Nikon). The number of Iba-1 positive cells was counted with Image Pro Plus 6.0 Software (MediaCybernetics, Bethesda, USA).

### Enzyme Linked Immunosorbent Assay

The right hemisphere of brain was separated for the determining of the levels of inflammatory factors by a ELISA kits according to the instructions (TNF-α from Diaclone Research, Besancon, France; IL-1β, IL-6 from BioSource Europe SA, Nivelles, Belgium).

### Quantitative Real-Time Polymerase Chain Reaction (RT-qPCR)

The quantification of mRNA levels of inflammatory factors was performed by RT-qPCR as previously described [38]. The right hemisphere of brain (perforation side) was obtained at 24 h after SAH and total RNA from the cortex was isolated using TRIzol (Invitrogen, USA) with the PureLink RNA Mini Kit (Invitrogen, USA) as instructions. The purity and concentration of RNA were tested by spectrophotometric analysis (OD 260/280) and agarose gel electrophoresis. Then RNA was reverse-transcribed to obtain cDNA using SuperScript II Reverse Transcriptase (Invitrogen, USA). The reaction was ended by heating at 70 °C for 10 min. RT-qPCR was performed using the CFX96 Real-time PCR detection system (Bio-Rad, USA), applying real-time SYBR Green PCR technology. The reaction mixtures (25 μl) contained 12.5 μl SYBR Green, 9.5 μl nuclease-free water, 1 μl cDNA, 1 μl forward primer (10 μM) and 1 μl reverse primer (10 μM). The primers sequences were designed by the Genscript Biological Technology Company (Nanjing, China) according to the gene sequences reported in GenBank. The IL-1β primers were 50′ -CACCTCTCAAGCAGAGCACAG-30′ (forward) and 50′-GGGTTCCATGGTGAAGTCAAC-30′ (reverse). The IL-6 primers were 50′-TCCTACCCCAACTTCCAATGCTC-30′ (forward) and 50′-TTGGATGGTCTTGGTCCTTAGCC-30′ (reverse). The TNF-α primer were 50′-AAATGGGCTCCCTCTCATCAGTTC-30′ (forward) and 50′-TCTGCTTGGTGGTTTGCTACGAC-30′ (reverse). The GAPDH primers were 5′-ACAGCAACAGGGTGGTGGAC-3′ (forward) and 5′-TTTGAGGGTGCAGCGAACTT-3′ (reverse). After 95 °C for 1 min, 40 cycles were performed and each cycle contained a denaturation step at 95 °C for 15 s and an annealing step at 60 °C for 1 min. The melting curve which was measured immediately after amplification, showed a single product peak indicating the good product specificity. All samples were analyzed in triplicate. Data were analyzed using the Line-Gene software. The mRNA expression levels were calculated with the 2-ΔΔCq method and normalized by quantity of GAPDH mRNA.

### Statistic Analysis

All data were presented as means ± standard error of the mean (SEM) and SPSS 12.0 was used for statistical analysis of the results. Data were subjected to one-way analysis of variance (ANOVA) followed by the LSD and Dunnett’s post-hoc test for multiple comparisons. The difference of mortality rate was analyzed by Chi square test. A value of p < 0.05 was considered as statistical significance.

## Results

### Effects of EP4 Receptor on SAH Grade, Mortality and Neurological Score

**Fig. 1:**
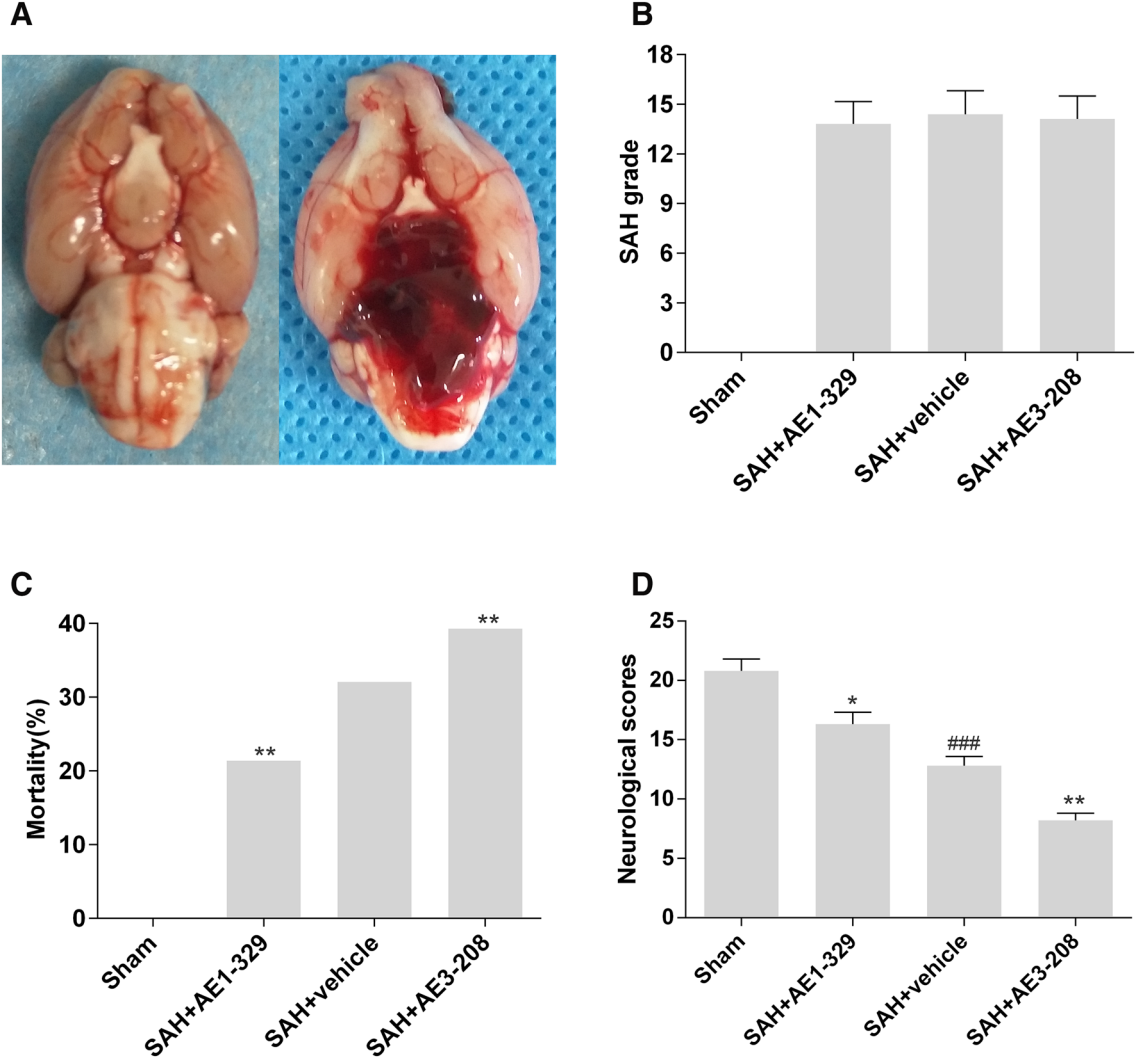
Effects of EP4 receptor on SAH grade, mortality and neurological score 24 h after SAH. Representative images showed rat brains of sham and SAH groups (**a**). EP4 modulation did not affect the SAH grade (n = 12) (**b**). The activation of EP4 by AE1-329 decreased mortality (n = 28) and attenuated neurological deficits (n = 12) 24 h after SAH while the inhibition of EP4 by AE3-208 showed opposite effects (**c, d**). Data were expressed as mean ± SEM (^###^p < 0.001 vs. Sham, * p< 0.05 vs. SAH treated with vehicle, **p < 0.01 vs. SAH treated with vehicle)

Twenty-four hours after SAH, the SAH grade of SAH group treated with vehicle (14.4 ± 1.41) did not significantly differ from those of treated with EP4 receptor specific agonist AE1-329 (13.8 ± 1.36) or antagonist AE3-208 (14.1 ± 1.38), suggesting the modulation of EP4 receptor by AE1-329 or AE3-208 did not affect the bleeding levels (Fig. 1b).

There are no animals of sham group died after surgery. The mortality of the SAH treated with vehicle was 32.1% (9 of 28 rats). The mortality of the SAH treated with AE1-329 and AE3-208 was 21.4% (6 of 28 rats) and 39.3% (11 of 28 rats) respectively (Fig. 1c).

The neurological score was significantly decreased in the SAH group treated with vehicle (12.8 ± 1.3) compared with the sham group (20.8 ± 1.5). AE1-329 administration increased neurological score (16.3 ± 1.6) compared with SAH treated with vehicle, while AE3-208 showed opposite effects (9.2 ± 0.9) (Fig. 1d).

### Effects of EP4 Receptor on Brain Edema and BBB Permeability After SAH

**Fig. 2:**
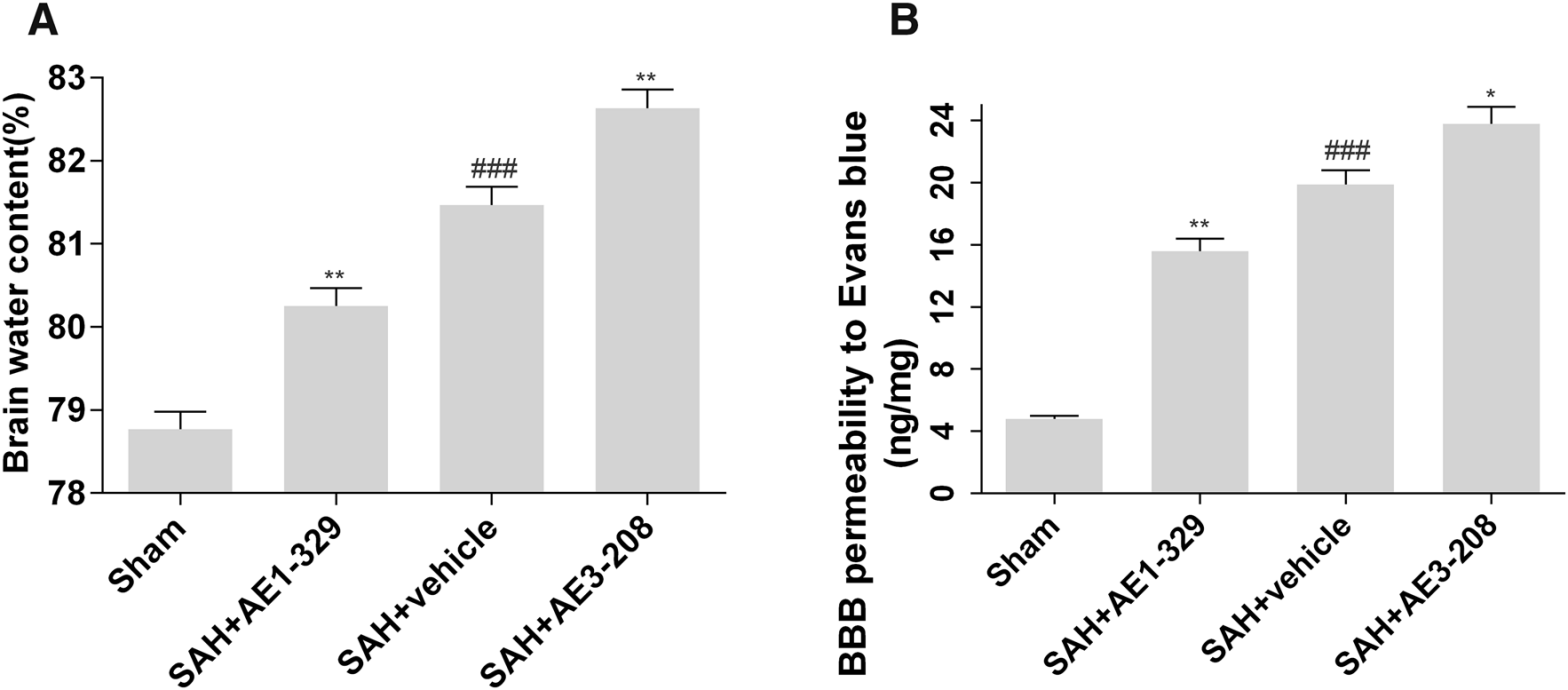
Effects of EP4 receptor on brain edema and BBB permeability after SAH. The activation of EP4 by AE1-329 decreased brain water content and BBB permeability to Evans blue 24 h after SAH while the inhibition of EP4 by AE3-208 showed opposite effects (**a, b**). Data were expressed as mean ± SEM (n = 12, ^###^p < 0.001 vs. Sham, *p < 0.05 vs. SAH treated with vehicle, **p < 0.01 vs. SAH treated with vehicle)

Twenty-four hours after SAH, brain water content of SAH group treated with vehicle (81.47 ± 0.22%) was significantly increased compared with sham group (78.77 ± 0.21%). AE1-329 decreased brain water content (80.25 ± 0.22%) while AE3-208 increased the brain water content (82.63 ± 0.23%) (Fig. 2a). Rats of SAH group treated with vehicle showed a significant increase in BBB permeability to Evans blue (19.9 ng/mg protein) indicating an early damage of BBB after SAH. AE1-329 improved the BBB damage as demonstrated by the decrease of Evans blue extravasation (15.6 ng/mg protein) while AE3-208 showed opposite effects (23.8 ng/mg protein) (Fig. 2b).

### Effects of EP4 Receptor on Neuronal Apoptosis After SAH

**Fig. 3:**
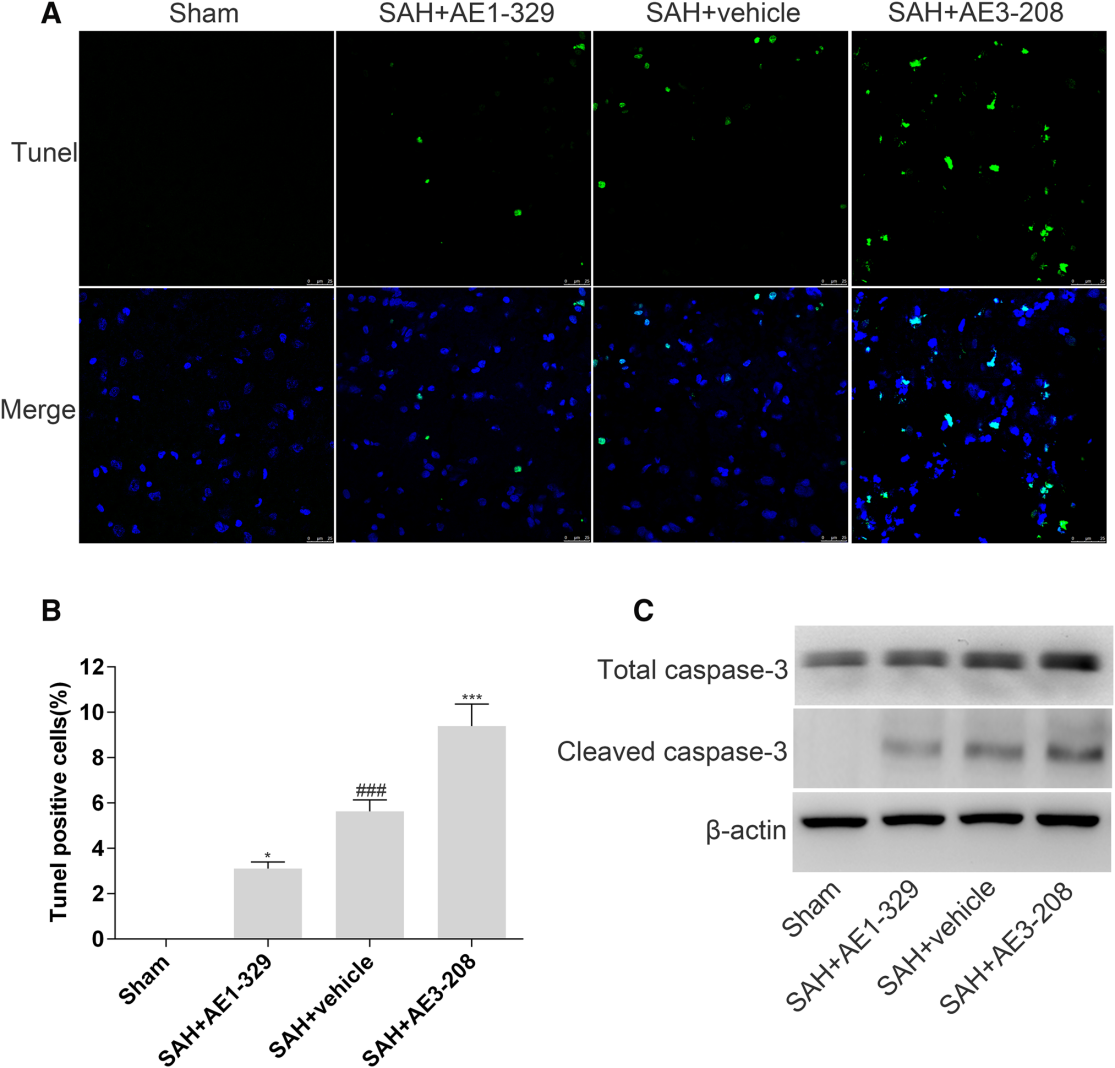
Effects of EP4 receptor on neuronal apoptosis after SAH. TUNEL study showed the activation of EP4 by AE1-329 attenuated neuronal apoptosis 24 h after SAH while the inhibition of EP4 by AE3-208 showed opposite effects (**a, b**). Western blot analysis showed AE1-329 decreased the expression of cleaved caspase-3 24 h after SAH and AE3-208 showed opposite effects (**c**). Nuclear morphology was indicated by DAPI staining and DNA breaks were detected by TUNEL analyses. Bar: 25 µm. Data were expressed as mean ± SEM (n = 8, ^###^p < 0.001 vs. Sham, *p < 0.05 vs. SAH treated with vehicle, ***p < 0.001 vs. SAH treated with vehicle)

**Fig. 4:**
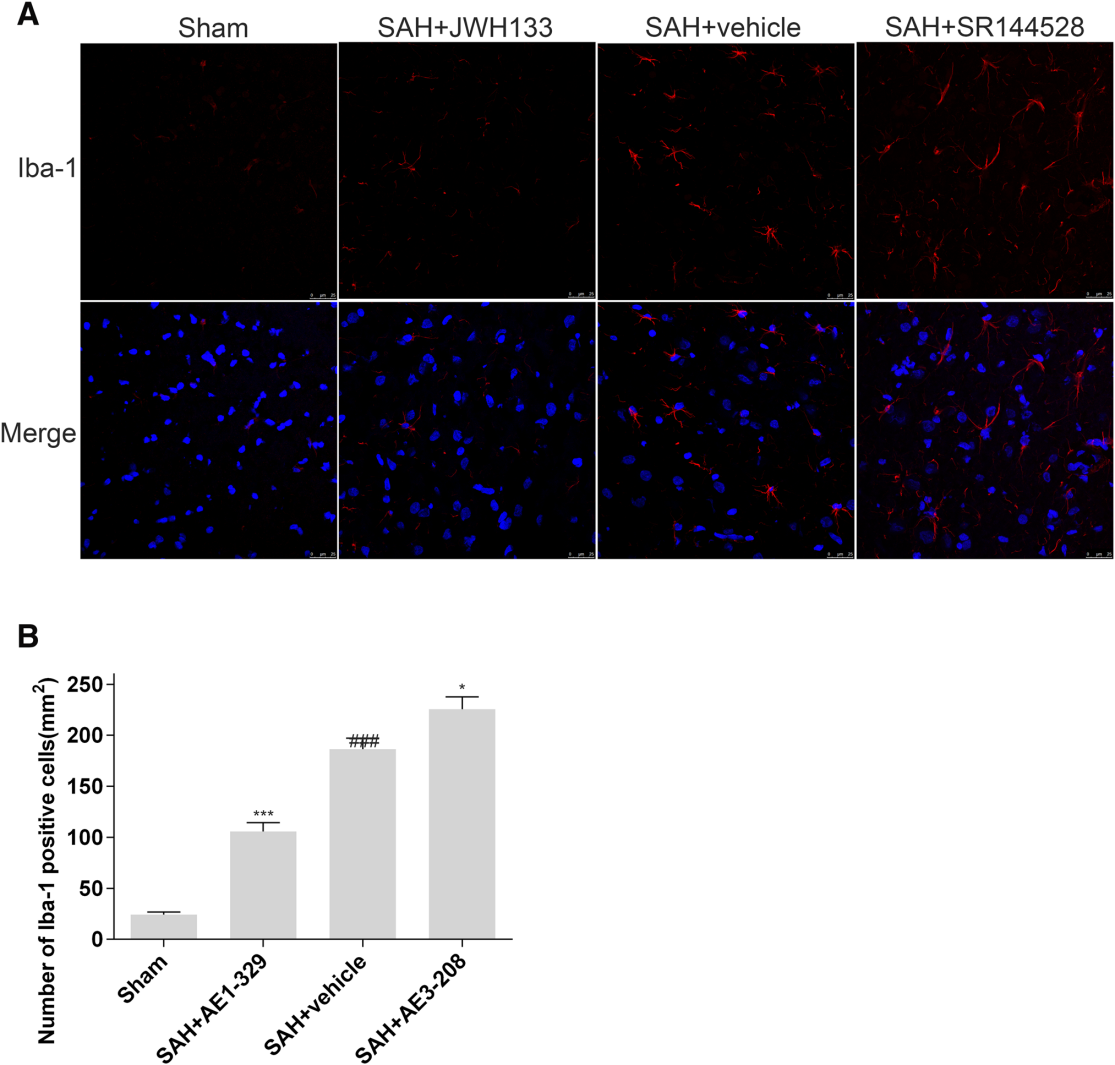
Effects of EP4 receptor on the activation of microglia after SAH. Iba-1 staining for microglias showed activation of EP4 by AE1-329 attenuated the number and activation of microglias in the brain 24 h after SAH while inhibition of EP4 by AE3-208 showed opposite effects (**a, b**). Microglia was indicated by Iba-1 staining and nuclear was indicated by DAPI staining. Bar: 25 µm. Data were expressed as mean ± SEM (n = 8, ^###^p < 0.001 vs. Sham, *p < 0.05 vs. SAH treated with vehicle,***p < 0.001 vs. SAH treated with vehicle)

Twenty-four hours post SAH, about 5.64 ± 0.51% cells of SAH group treated with vehicle showed apoptosis (Fig. 4a, b). AE1-329 significantly mitigated cellular apoptosis (3.12 ± 0.29%) while AE3-208 aggravated cellular apoptosis (9.39 ± 0.97%) (Fig. 3a, b). The expression of cleaved caspase-3 in SHA group also increased dramatically compared with that of sham group, which was downregulated by AE1-329 and upregulated by AE3-208 (Fig. 3c).

### Effects of EP4 Receptor on the Activation of Microglia After SAH

After SAH, microglia transform from the resting status to highly activated status. The hallmarks of activated microglia include morphological changes, production of pro-inflammatory cytokines and enhanced proliferation [39–41].

In sham group, the density of Iba-1 positive cells was 24.3 ± 2.47/mm^2^. Twenty-four hours post SAH, the microglias in SAH treated with vehicle group increased in number (186.5 ± 10.7/mm^2^) and became activated, altering their morphology with upregulation of Iba-1 (Fig. 4a, b). AE1-329 significantly attenuated Iba-1 positive microglia number (105.8 ± 8.5/mm^2^) and it^’^s activation, while AE3-208 showed opposite effects (225.5 ± 12.4/mm^2^) (Fig. 4a, b).

### Effects of EP4 Receptor on the Levels of Inflammatory Cytokines After SAH

**Fig. 5:**
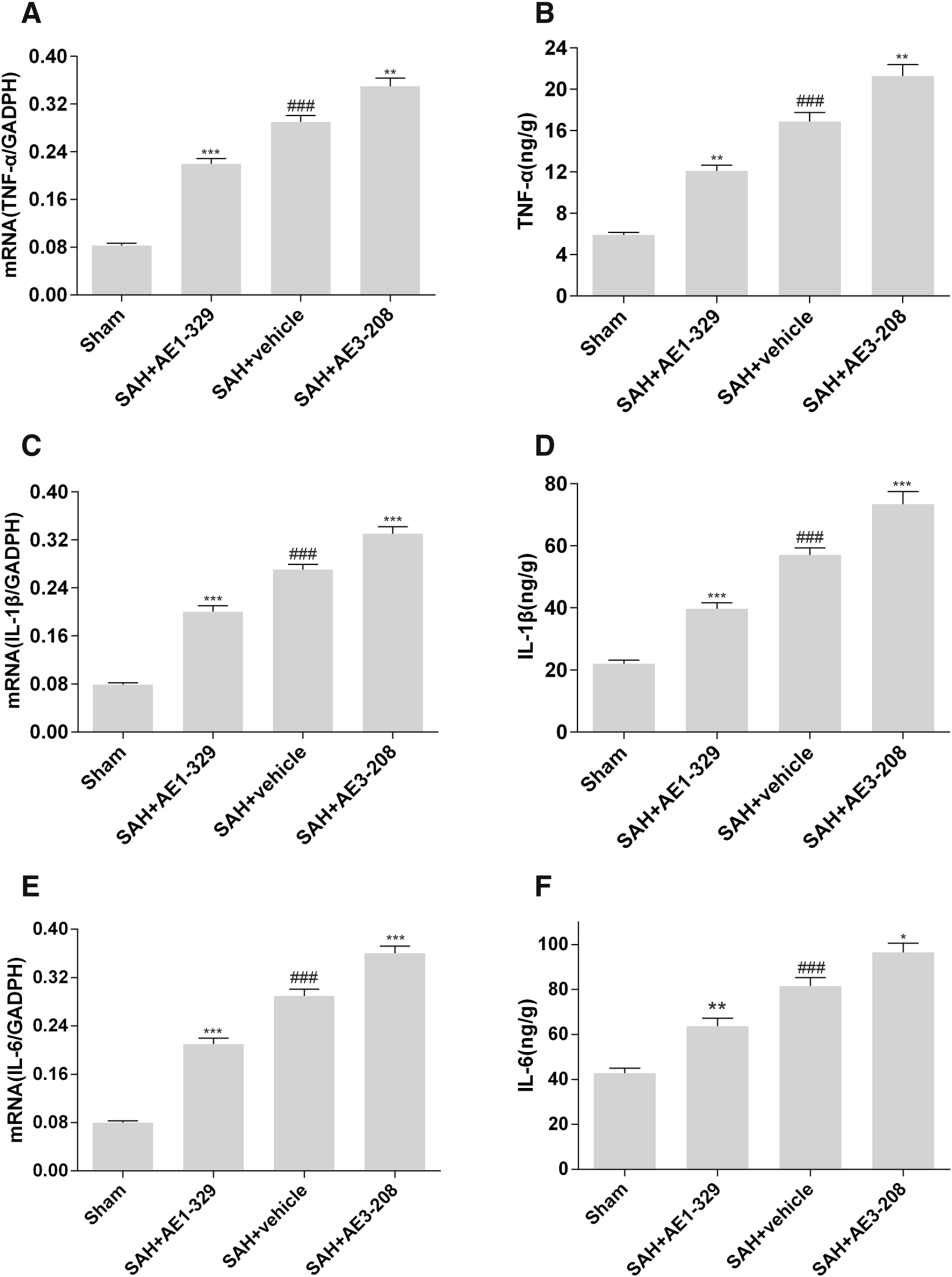
Effects of EP4 receptor on the levels of inflammatory cytokines after SAH. RT-qPCR analysis showed EP4 activation by AE1-329 reduced the mRNA expression of TNF-a IL-1β and IL-6 while inhibition of EP4 by AE3-208 increased the mRNA expression (**a, c, e**). Enzyme linked immunosorbent assay showed activation of EP4 by AE1-329 attenuated the levels of inflammatory cytokines while inhibition of EP4 by AE3-208 showed opposite effects (**b, d, f**). Data were expressed as mean ± SEM (n = 8, ^###^p < 0.001 vs. Sham, *p < 0.05 vs. SAH treated with vehicle, **p < 0.01 vs. SAH treated with vehicle, ***p < 0.001 vs. SAH treated with vehicle)

To further explore the effects of EP4 receptor activation on the neuroinflammation after SAH, we measured the mRNA and protein levels of pro-inflammatory cytokines TNF-a, IL-1β and IL-6 in the cortex at 24 h after injury. RT-qPCR analysis showed AE1-329 reduced the mRNA expression of TNF-a, IL-1β and IL-6 while AE3-208 increased the mRNA expression (Fig. 5a, c, e).

Enzyme linked immunosorbent assay showed, compared with the sham group (5.9 ± 0.27 ng/g in TNF-α, 22.1 ± 1.1 ng/g in IL-1β and 42.8 ± 2.2 ng/g in IL-6 respectively), the levels of inflammatory cytokines significantly increased in the SAH treated with vehicle group (16.9 ± 0.85 ng/g in TNF-α, 57.0 ± 2.3 ng/g in IL-1β and 81.4 ± 3.9 ng/g in IL-6 respectively) 24 h after SAH. AE1-329 downregulated the levels of inflammatory cytokines (12.1 ± 0.54 ng/g in TNF-α, 39.7 ± 1.9 ng/g in IL-1β and 63.7 ± 3.5 ng/g in IL-6 respectively) while AE3-208 showed opposite effects (21.3 ± 1.1 ng/g in TNF-α, 73.4 ± 4.1 ng/g in IL-1β and 94.5 ± 4.6 ng/g in IL-6 respectively) (Fig. 5b, d, f).

### Effects of EP4 Receptor on the Expression of p-eNOS

**Fig. 6:**
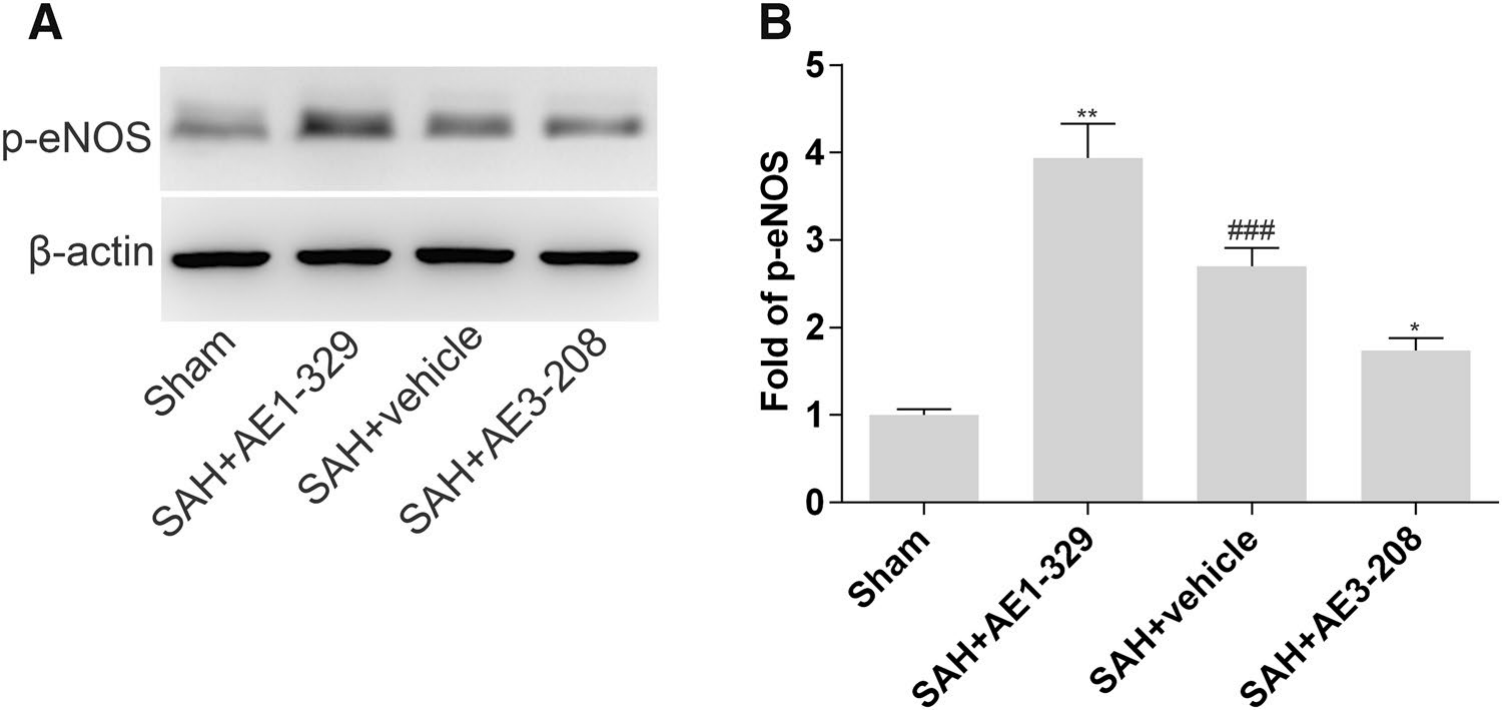
Effects of EP4 receptor on the expression of p-eNOS. Western blot analysis showed AE1-329 increased the expression of Ser1177 p-eNOS 24 h after SAH and AE3-208 showed opposite effects (**a, b**). Data were expressed as mean ± SEM (n = 8, ^###^p < 0.001 vs. Sham, *p < 0.05 vs. SAH treated with vehicle, **p < 0.01 vs. SAH treated with vehicle)

Western blot analysis showed Ser1177 p-eNOS expression increased 24 h after SAH (2.70 folds higher than sham group) (Fig. 6a, b). Compared with SAH treated with vehicle group, AE1-329 increased the expression of Ser1177 p-eNOS (3.94 folds higher than sham group) and AE3-208 showed opposite effects (1.74 folds higher than sham group) (Fig. 6a, b).

## Discussion

In this study we present the neuroprotective effects induced by EP4 receptor activation on EBI after SAH. Our results showed that the activation of EP4 by AE1-329 significantly improved neurological dysfunctions, reduced mortality, attenuated brain edema and BBB damage 24 h after SAH. In addition, EP4 activation downregulated the microglial activation, reduced the pro-inflammatory cytokine concentrations such as TNF-α, IL-1β and IL-6 in cortex and increased the Ser1177 p-eNOS expression 24 h after SAH. Moreover, EP4 activation decreased the number of TUNEL-positive cells and active caspase-3 in cortex 24 h after SAH. These findings suggested that EP4 activation protected brain from SAH-induced EBI. The contrary effects induced by EP4 specific antagonist AE3-208 further contributed to determine the roles of EP4 in EBI after SAH.

The pathophysiologic mechanisms related to EBI are under investigating. Increased intracranial pressure (ICP), reduced cerebral blood flow (CBF) and cerebral perfusion pressure (CPP), BBB damage, brain edema, brain swelling and dysfunction of autoregulation, all of which stem from the initial bleeding in SAH, constitute the pathophysiological variables occurring at the EBI period [42]. Among them, the most immediate event following SAH is an arrest in intracranial circulation caused by a peak of ICP which can reach to the level of mean arterial blood pressure shortly after SAH [43]. This temporary circulatory arrest results in a hypoxic state which leads to the apoptosis of neurons and glias due to the energy failure, and initiates the cascade of events leading to cytotoxic edema [42]. Ischemia also causes apoptosis of cells that constitute the BBB, which results in the increased diffusion of serum from the vascular lumen into cerebral tissues and eventually the vasogenic edema [44]. SAH also activates astrocytes and microglias, and upregulates the production and secretion of pro-inflammatory cytokines [45, 46]. Pro-inflammatory cytokines enhance brain edema through the disrupting of BBB and induce neuronal apoptosis, and therefore directly contribute to early brain damage [10, 47].

Our data indicated that the activation of EP4 receptor by AE1-329 improved brain edema, BBB damage and neurological outcomes. These results are consistent with some previous studies demonstrating that the activation of EP4 receptor shows neuroprotective effects on other CNS insults such as cerebral ischemia [23, 24], hypoxic-ischemic encephalopathy (HIE) [25], neurotoxicity induced brain injury [26] and Alzheimer^’^s disease [21].

The mechanisms underlying these EP4-induced neuroprotective effects were complicated and multiple cell types, including microglias, neurons and endothelial cells might be involved. Our findings indicated that the pharmacologic activation of EP4 receptor elicited the downregulation of microglial activity, the mRNA and protein expression of inflammatory cytokines. The production of pro-inflammatory cytokines is one of the hallmarks of microglial activation. In our study, we did not explored how SAH-induced production of TNF-a IL-1β and IL-6 was regulated by EP4 receptor. The suppression of microglial activation might account for the reduction of the pro-inflammatory cytokines. Going further, the activation of nuclear factor-kappaB (NF-κB) is required for the generating of TNF-a, IL-1β and IL-6 in microglia [48]. Woodling et al. have found that EP4 signaling broadly represses the activation of target genes for interferon regulatory factors (IRFs) and NF-κB, which are central transcription factors regulating the microglial response to Aβ_42_ stimulation [21]. The similar NF-κB suppression after EP4 activation has also been reported by Shi et al. in a brain innate immunity model induced by lipopolysaccharide (LPS) [22].

In addition to anti-inflammatory effects, we also found the selective activation of EP4 significantly decreased the number of TUNEL positive cells and level of active caspase-3 after SAH. This should not be entirely ascribed to the suppression of neuroinflammatory reaction after EP4 activation, as EP4 receptors also locate on neurons and their activation by AE1-329 have shown a neuroprotective effect on brain ischemia [23].

We also found the upregulation of Ser1177 p-eNOS expression after AE1-329 administration. The endothelial EP4 receptor which expresses at a very low level in normal condition markedly upregulates in vasculature under the stimulation of ischemia and systemical administration of AE1-329 greatly elevate protein levels of eNOS and activated phospho-Ser1177 eNOS in mouse cerebral microvessels [23]. The activation of eNOS and increased NO production result in cGMP dependent smooth muscle cell relaxation and increased cerebral blood flow [49]. Endothelial EP4 activation by AE1-329 might directly inhibit the increase in BBB permeability in mouse experimental autoimmune encephalomyelitis [31].

Another neuroprotective mechanism initiated by EP4 activation in this study might involve the peripheral inflammatory cells including neutrophils, macrophages and lymphocytes. Transmission of inflammatory cells from periphery blood to CNS has been demonstrated in the clinical and animal studies of SAH, which can result in not only the neuroinflammatory aggravation but also the early neuronal loss and BBB damage [50, 51]. Peripheral administration of the EP4 specific agonist AE1-329 downregulates the secreted levels of pro-inflammatory cytokines and chemokines from macrophages and neutrophils [22, 52].

Taken together, in present study, EP4 activation appears to have a multiple beneficial properties for EBI after SAH. However, the precise mechanisms of the neuroprotective effects of EP4 activation on EBI after SAH remain unclear and further studies exploring the underlying mechanisms of EP4 are required in future.

## Conclusion

This study is the first one to explore the effects of EP4 receptor activation on EBI after experimental SAH. We find the activation of EP4 receptor by AE1-329 improves neurological dysfunctions, and ameliorates brain edema and BBB damages after SAH. Meanwhile activation of EP4 receptor suppresses microglial activation, the protein and mRNA levels of pro-inflammatory cytokines TNF-a, IL-1β and IL-6 and increases Ser1177 p-eNOS expression. Furthermore, activation of EP4 receptor attenuates cellular apoptosis through downregulating active caspase-3. Our results suggest EP4 receptor may be a promising potential target for SAH therapy.
